# Investigating the Influence of *n*-Heptane versus *n*-Nonane upon the Extraction of Asphaltenes

**DOI:** 10.1021/acs.energyfuels.2c01168

**Published:** 2022-08-01

**Authors:** Latifa K. Alostad, Diana Catalina Palacio Lozano, Benedict Gannon, Rory P. Downham, Hugh E. Jones, Mark P. Barrow

**Affiliations:** † Department of Chemistry, University of Warwick, Coventry CV4 7AL, United Kingdom; ‡ Molecular Analytical Sciences Centre for Doctoral Training, University of Warwick, Coventry CV4 7AL, United Kingdom

## Abstract

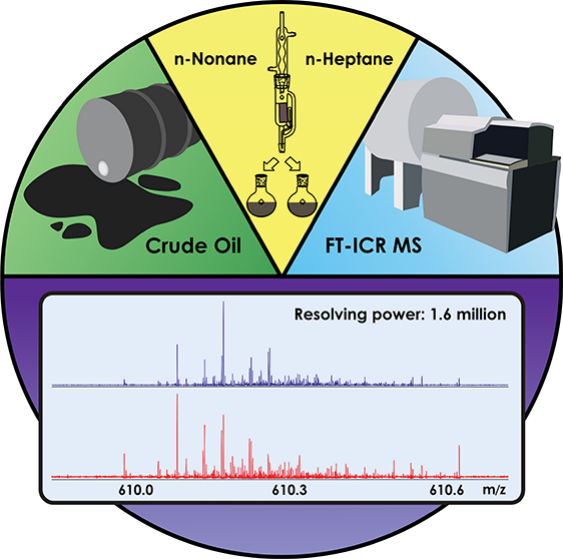

The composition of asphaltenes is of interest due to the challenges they pose for industry and their high complexity, encompassing a range of heteroatom contents, molecular weights, double bond equivalents (DBEs), and structural motifs. They are well-known for aggregating above critical concentrations, hindering the upstream and downstream processes. Asphaltenes are defined by solubility, as they are insoluble in light paraffins such as *n*-heptane and soluble in aromatic solvents such as toluene. Today, enormous efforts are being invested into the characterization of asphaltenes to shed light into their structural profiles to benefit the petroleum industry and environmental sustainability. Fourier transform ion cyclotron resonance mass spectrometry (FT-ICR MS) provides molecular level analysis with unparalleled mass resolving power and mass accuracy, which is vital for the characterization of inherently complex crude oils and their asphaltene fractions. The aim of this research is to elucidate and compare the compositional profiles of asphaltene fractions of two petroleum samples, fractioned through two approaches: using *n*-heptane, as is typical practice, and *n*-nonane, for the purpose of testing extraction using higher molecular weight alkanes. The results highlight that the choice of solvents does indeed influence the accessibility of different species and therefore changes the observed molecular profiles of the extracted asphaltenes. *n*-Heptane afforded broader contributions of different heteroatomic classes and greater carbon number ranges of the observed components; the DBE distribution vs carbon number profiles were different, where the extracts produced using *n*-nonane displayed a greater prevalence of lower DBE species.

## Introduction

Asphaltenes,^1^ a fraction of petroleum components defined by solubility, are soluble in aromatic solvents such as toluene and insoluble in low-molecular-weight *n*-alkanes, such as *n*-hexane or *n*-heptane.^2−4^ Asphaltenes are nondistillable and must therefore be obtained by fractionating crude oil using solvent-based methods,^5^ which commonly involve precipitation with *n*-heptane.^6^ Other solvents such as benzene, carbon disulfide, and chloroform were used to separate asphaltenes; however, they may not effectively dissolve the *n*-heptane components especially for heavier petroleum types.^4^ The high viscosity of asphaltenes, arising from their aromatic compositions^7,8^ as well as asphaltene deposition due to flocculation results in clogged pipelines, can hinder the oil production process. Presently, this is managed by control of pressure, temperature, and flow rate, though these methods can be inefficient. Alternative solutions could be sought by achieving greater understanding of asphaltene mixtures, including their chemical compositions and solubility characteristics, enabling application of additives such as dispersants.

Asphaltene composition has been investigated and debated for over five decades. Some of these debates have resulted in a new understanding of the molecular structures of asphaltenes, particularly with respect to their molecular weights. Advanced mass-spectrometry-based methods concluded that asphaltenes inhibit mass ranges of 250–1200 g mol^–1^ rather than the misconception of this fraction having molecular masses in the range of thousands of daltons.^9^ Some debates remain ongoing; there is significant evidence that the “island” (a single aromatic core with radial alkyl chains) structure dominates over the “archipelago” (multiple aromatic cores, connected by alkyl chains) structure in mixture,^10^ although the extent to which this holds in different samples is still being investigated.^11−14^

Other features of asphaltenes include a diversity in heteroatom classes and complexity of chemical composition. These factors make conclusions about composition by low-resolution instruments difficult or impossible,^15^ notably by gas chromatography coupled with mass spectrometry (GC-MS) where the full double bond equivalent (DBE) range of an asphaltene mixture was found to be inaccessible.^16^ To this end, Fourier transform ion cyclotron resonance mass spectrometry (FT-ICR MS) has become well-established as playing a vital role for these analyses, due to its ultrahigh resolving power and high mass accuracy.^17−20^ In turn, the capabilities of advanced mass spectrometry methods have led to the field of research known as “petroleomics”.^21^ With the benefits of ultrahigh resolving power and mass accuracy, the many thousands of molecules within complex mixtures can be determined and subsequently categorized by their heteroatom class, carbon number, and DBEs. As analytical approaches become more advanced, the complexity of such samples becomes more fully appreciated; recent work has revealed that petroleum-related samples may comprise hundreds of thousands of unique molecular formulas,^22,23^ and each of these formulas will have many associated isomers.

Due to asphaltenes being defined by solubility class, various nonaromatic solvents and solvent blends are used in industry to precipitate asphaltenes from crude oils and bitumen.^24^ However, solvent choice and temperature may both affect the yield and properties of the precipitated asphaltene fraction, with yield being further influenced by the dilution ratio for a given solvent and the solvent–oil contact-time.^24^

Various models have been proposed for predicting asphaltene precipitation, based on the solubility parameter concept,^25,24^ which relies on the assumption of two crude oil phases—the asphaltenes and the remaining oil components, known as maltenes—and their phase equilibria.^25^ Understanding the insolubility of asphaltenes in *n*-alkanes can be approached in terms of known structures and associated intermolecular forces.^5,24^ Molecular size, increased potential for hydrogen bonding due to high heteroatom count, interactions between acidic and basic structural moieties (such as carboxylic acid groups and pyridine rings), and the phenomenon of aromatic core π-stacking are among the interactions that have been investigated in the literature.^5,16,26−30^ While π-stacking emerged as the likely dominant factor responsible for asphaltene aggregation,^27,28^ with heteroatom-based interactions thought to have no impact on aggregation or solubility, recent work by Chacón-Patiño et al.^31−33^ highlighted the significance of polyoxygenated species in asphaltene solubility, which aligns with the concepts postulated in the earlier Boduszynski continuum theory.^27^

Another model, first developed by Yen^34^ and then adapted to incorporate a modern understanding of the mixture by Mullins into the modified Yen model,^35,36^ has also been formulated. This model combines the new research suggesting the dominant island structure of asphaltenes, with an archipelago minority, and its aggregation behavior (“nanoaggregation”) with that of the original model to form the basis for a range of future investigations. Using fragmentation techniques, such as infrared multiphoton dissociation (IRMPD), recent studies have revealed that the predominant motifs are sample dependent.^31,32^

A supramolecular assembly model was proposed by Gray et al.^937^ to explain the aggregation of asphaltenes with contributions from noncovalent bonds. The complex and diverse composition of asphaltenes can encourage intermolecular interactions such as π–π stacking, hydrogen bonding, acid–base, van der Waals, and electrostatic interactions. The combination of weak forces connecting the building blocks results in strongly accumulated aggregates.^938,37^ From a research perspective, the preparation of asphaltenes must be undertaken with care, as traditional *n*-alkane approaches can generate fractions contaminated with maltenes and microcrystalline waxes.^39^ Once isolated, more detailed analyses can be facilitated by further dividing the highly complex asphaltenes into a series of finer fractions,^38^ leading to improved characterization.^6,39,40^ Examples of fractionation approaches include sequential elution fractionation performed in an inert column, solvent extraction such as via Soxhlet,^41^ differential precipitation in solvent mixtures,^27^ column chromatography, ultracentrifugation, and supercritical fluid extraction.^6^

In petroleomics, nonpolar species are frequently ionized using a variety of ion sources, including atmospheric pressure chemical ionization (APCI)^43,44^ and photon-based ionization sources such as atmospheric pressure laser ionization (APLI)^42^ and atmospheric pressure photoionization (APPI).^43^ This work focuses on the use of APPI, as it successfully ionizes a range of molecular compositions including polycyclic aromatic hydrocarbons (PAHs) for the successful detection of asphaltenes by mass spectrometry.^45^

The aforementioned recent work by Chacón-Patiño et al. deployed Soxhlet extraction to subfractionate an island-enriched asphaltene sample with *n*-heptane and an *n*-heptane/toluene differential precipitation approach to subfractionate an archipelago-enriched asphaltene sample.^27^ The subfractions were subsequentially analyzed for molecular characterization via an APPI source coupled to FT-ICR MS, with operation in positive-ion mode.^27^ Results showed that polyoxygenated compounds, especially the sulfur-containing classes (e.g., O_2_S_2_) with lower DBE values, were concentrated in the more insoluble asphaltene subfractions. It is hence very likely that the occurrence of such compounds in the more insoluble asphaltene subfractions relates to hydrogen bonding interactions.^27^

The following study examines the use of two *n*-alkane solvents for the extraction of asphaltenes. Two Middle Eastern petroleum samples (Table 1) were selected for comparison, and each sample was subjected to two, parallel extraction processes. One extraction involved the use of *n*-heptane, which is traditionally used, while the other extraction process involved *n*-nonane to shed the light on asphaltenes extracted with higher molecular weight hydrocarbons and hence understand the compositional profile of the two petroleum samples and two extraction methods; a total of four samples resulted. The role of the extraction solvent was found to influence the observed heteroatom class profiles and the compositional boundaries that can be accessed, with respect to the number of components detected and their molecular weight ranges.

**Table 1:** Properties of Unfractionated Crude 1 and Unfractionated Crude 2

parameters	method	Crude 1	Crude 2
density at 15 °C, g/mL	D5002/IP365	0.9632	0.9643
gravity °API	D1250/IP200	15.25	15.09
asphaltenes, % wt	Cosmo Analyzer D5708	7.0	5.3
sulfur, % wt	D4294	4.82	5.05

## Material and Methods

### Automated Soxhlet Extractions

Samples of 10 g of two heavy Middle Eastern crude oils (Table 1), referred to here as Crude 1 and Crude 2, were diluted with 300 mL of *n*-heptane (HPLC grade, Fisher Scientific, Hertfordshire, United Kingdom) and kept for 24 h. The mixture was subsequently filtered using a 0.5 μm ash 40 M filter paper. The filtrate and the filter paper were then transferred for automated Soxhlet extraction (Buchi, BUCHI UK Ltd.) with ∼150 mL of *n*-heptane to be heated according to the heating program of the chosen solvent for 24 h. Finally, a different heating program was applied in combination with toluene (Chromasolv grade, Honeywell Riedel-de Haën Seelze GmbH, Hanover, Germany).

### Manual Soxhlet Extractions

Of the two heavy Middle Eastern samples, Table 1, 10 g each were separately diluted with 300 mL of *n*-nonane (99% purity, Fisher Scientific, Hertfordshire, United Kingdom) and stored for 24 h. Next, each mixture was filtered using a 0.5 μm ash 40 M filter paper. The filtrate and the filter paper were then transferred to a manual Soxhlet extraction setup with ∼150 mL of *n*-nonane for 24 h at ∼200 °C. Afterward, the solvent was changed to toluene to dissolve the asphaltene fraction and heated at ∼280 °C for 24 h.

### FT-ICR MS

Asphaltene fractions from both Soxhlet extraction experiments were dissolved in toluene to a concentration of 0.04 mg mL^–1^, where the concentration was limited in order to avoid aggregation of asphaltenes. Magnitude mode mass spectra were acquired using a modified 15 T solariX XR Fourier transform ion cyclotron resonance (FT-ICR) mass spectrometer (Bruker Daltonik GmbH, Bremen, Germany), coupled to an APPI II source, which was operated in positive-ion mode. Data were acquired using the same instrument parameters for all samples. The following parameters were used to acquire the data: drying gas temperature of 200 °C, flow rate of 4 L min^–1^, nebulizer gas pressure of 2.5 bar, krypton lamp producing photons with energies of 10.0 and 10.6 eV, direct infusion using a syringe pump flow rate of 600 μL min^–1^, and 8 M data set sizes. The mass range detected was *m*/*z* 250–1200 with each data set produced from 400 summated scans. Following acquisition, FTMS Processing 2.3 (Bruker Daltonik GmbH, Bremen, Germany) was used offline to convert the magnitude mode data to absorption mode with half Hanning apodization (“Kilgour” option). The data were then internally calibrated using homologous series of the S_1_ class and analyzed using DataAnalysis 5.0 (Bruker Daltonik GmbH, Bremen, Germany). The data were transferred to Composer 1.5.6 (Sierra Analytics, Modesto, CA, USA) for assignment of molecular formulas based upon homologous series present within the asphaltene samples. During the assignment process, the following maxima were used for each element: C 200, H 1000, S 5, N 3, and O 3. Assignments of molecular formulas to peaks were inspected afterward for accuracy and reproducibility; as an example, the composition of C_45_H_68_S_1_ was assigned to the peak observed in each data set at *m*/*z* 640.50363. Averaged values of the aromaticity index (AI), modified aromaticity index (AI_mod_), and DBEs for the four samples are listed in Table S1 of the Supporting Information. Finally, in-house software known as KairosMS^46^ (University of Warwick, Coventry, UK) was used for further data analysis and visualization.

### Elemental Analysis: CHNS

The CHNS elemental contributions of the *n*-heptane samples were analyzed by Exeter Analytical Ltd. (Coventry, UK). The elemental analysis of CHN was conducted using a CE440 Elemental Analyzer, while sulfur content was determined using inductively coupled plasma optical emission spectrometry (ICP-OES).

## Results and Discussion

**Figure 1:**
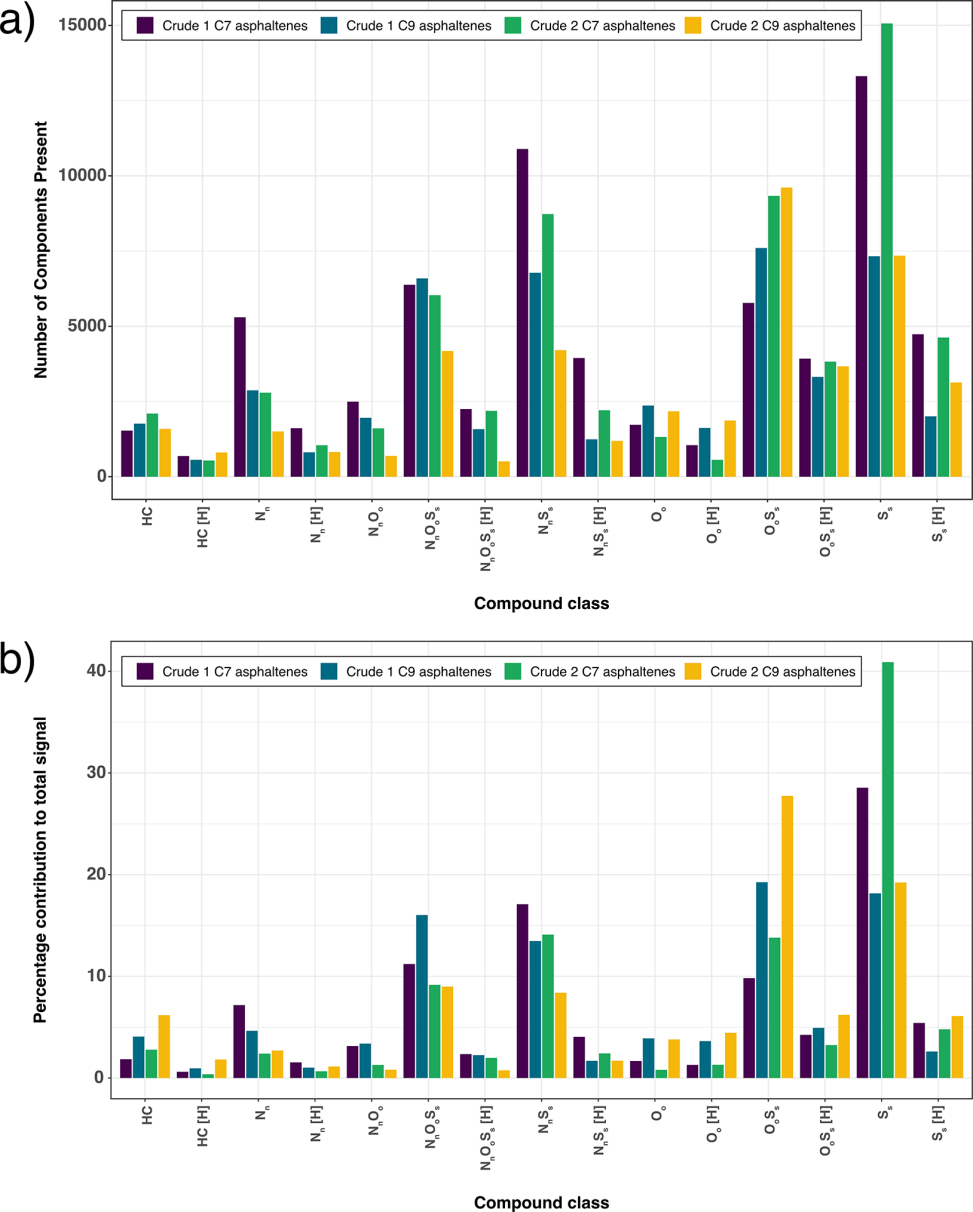
Comparison of grouped class contributions illustrating the (a) number of components present and (b) percentage contribution to the total signal of the four asphaltene samples analyzed by positive-ion APPI FT-ICR MS.

The asphaltene fractions obtained from Soxhlet extractions of Crude 1 and Crude 2 using *n*-heptane (C7) and *n*-nonane (C9) were compared to outline the differences in observed compositional profiles, arising from the use of different solvents. In the field of petroleomics, crude oil and its fractions can be categorized by means of heteroatoms, double bond equivalents (DBEs), and carbon numbers. Figure 1 illustrates grouped class categorization of heteroatoms contributing to the composition of the asphaltene fractions. Groups with [H] represent even-electron configurations, such as protonated species, whereas classes without this tag refer to radical ion species with odd-electron configurations. Both even-electron species and odd-electron species are expected to be observed using positive-ion mode APPI, as the ionization method can produce different ion types and ionize nonpolar species. By comparison, electrospray ionization (ESI), which is sometimes used for the analysis of petroleum, would preferentially ionize the polar species and produce even-electron ions (protonated species in positive-ion mode and deprotonated species in negative-ion mode).

Figure 1a shows the number of components observed for each group of heteroatom classes, while Figure 1b shows the same contributions as a function of signal intensity. The plots reveal the influence of the extraction solvents on promoting certain heteroatomic classes of the asphaltene mixtures. The use of *n*-heptane (referred to as C7 in the Figure) enhances the contributions from N_*n*_, N_*n*_[H], N_*n*_O_*o*_, N_*n*_O_*o*_S_*s*_[H], N_*n*_S_*s*_, N_*n*_S_*s*_[H], O_*o*_S_*s*_[H], S_*s*_, and S_*s*_[H] classes, when compared to the *n*-nonane fraction (referred to as C9 in the Figure). The N_*n*_S_*s*_, N_*n*_S_*s*_[H], S_*s*_, and S_*s*_[H] classes were most significantly influenced, as *n*-heptane efficiently extracted these classes from the crude oil sample. On the other hand, *n*-nonane promoted the contribution of O_*o*_ and O_*o*_[H] classes as well as the O_*o*_S_*s*_ class. When considering the effect upon signal intensity as shown in Figure 1b, the impact of *n*-heptane caused N_*n*_, N_*n*_S_*s*_, N_*n*_S_*s*_[H], S_*s*_, and S_*s*_[H] species to have stronger contributions, whereas the influence of *n*-nonane resulted in a stronger signal for the HC, HC[H], O_*o*_, O_*o*_[H], O_*o*_S_*s*_, and O_*o*_S_*s*_[H] compound classes. The S_*s*_ species were the most abundant group with significant contributions in the C7 fractions, with respect to both the count of the numbers of components and the signal contributions. Moreover, subtle compositional differences can be recognized, as asphaltenes separated from Crude 1 showed higher O_*o*_S_*s*_ content, whereas asphaltenes produced from Crude 2 displayed higher contributions of S_*s*_ and S_*s*_[H] classes. The N_*n*_, N_*n*_[H], N_*n*_O_*o*_, N_*n*_O_*o*_S_*s*_, N_*n*_O_*o*_S_*s*_[H], N_*n*_S_*s*_, N_*n*_S_*s*_[H], and O_*o*_ classes had a stronger contribution in Crude 1, while the HC, O_*o*_S_*s*_, S_*s*_, and S_*s*_[H] classes had a greater impact in Crude 2. The S_*s*_ classes were a noticeably more abundant contribution to Crude 2 than Crude 1, and these classes would most often represent various nonpolar sulfur-containing contributions, typically thiophenic species.

**Figure 2:**
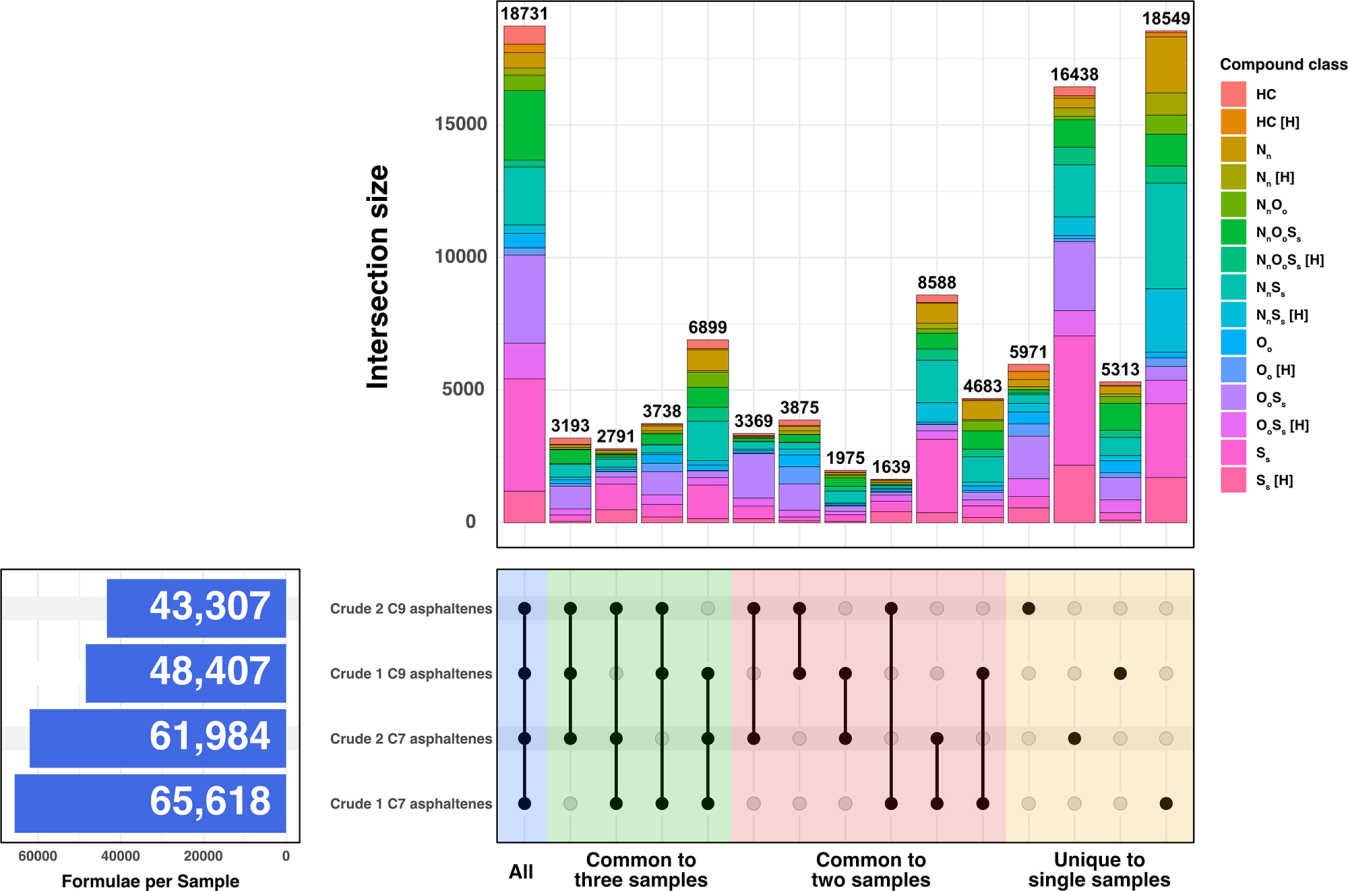
An UpSet chart displaying the common and unique compositions present in the four asphaltene samples.

To further illustrate the diversity of the chemical composition of the asphaltene extracts, an UpSet plot^949^ (Figure 2) was employed to represent the common and unique assignments when comparing the four samples. Each row represents a sample, where the presence of a black dot indicates an intersection and that the sample contributes to the bar chart above the dot. Where multiple samples are part of an intersection, the black dots are connected by a vertical black line. As an example, the region highlighted in blue has dots in front of all four sample names and a solid line connecting them, which demonstrates that the four samples have 18 731 species common between them; by comparison, the region highlighted in green shows three dots per comparison, indicating commonality between three specific samples each time. The bar chart at the top then shows the number of heteroatomic species present at that intersection, grouped by heteroatomic class. Where multiple samples are present for a given intersection, then the corresponding bar represents the number of species in common between those samples. If only one sample is present at an intersection, then this value instead represents the number of unique species to that sample. The four asphaltene samples had 18 731 compositions that were common between them with S_*s*_ being the largest group. O_*o*_S_*s*_ and N_*n*_S_*s*_O_*o*_ classes also have relatively high contributions to the common compositions for the four asphaltenes samples. It is also worth noting that 4683 species were found in common between the *n*-heptane and *n*-nonane extracts for Crude 1 with N_*n*_S_*s*_ and N_*n*_ species being highly abundant between them, while this number was 3369 species for the two Crude 2 extracts displaying the O_*o*_S_*s*_ class as the highly abundant class. Despite extracting asphaltenes from two samples within the same region using two different solvents, compositional differences are arising within Figure 2. Therefore, the range of compositions observed within a given class can vary between samples. *n*-Heptane extracts for both Crude 1 and Crude 2 exhibited a greater number of unique compositions (18 549 and 16 438, respectively) giving rise to two common classes S_*s*_ and S_*s*_[H] in comparison to the *n*-nonane extracts (5313 and 5971, respectively) with O_*o*_S_*s*_ constituents being the most abundant. The unique assignments of asphaltenes with the same origin but separated using different solvents, show that *n*-heptane can access S_*s*_, S_*s*_[H], and N_*n*_S_*s*_ species more than *n*-nonane, which targets O_*o*_S_*s*_ species, regardless of the compositional differences between the samples.

**Figure 3:**
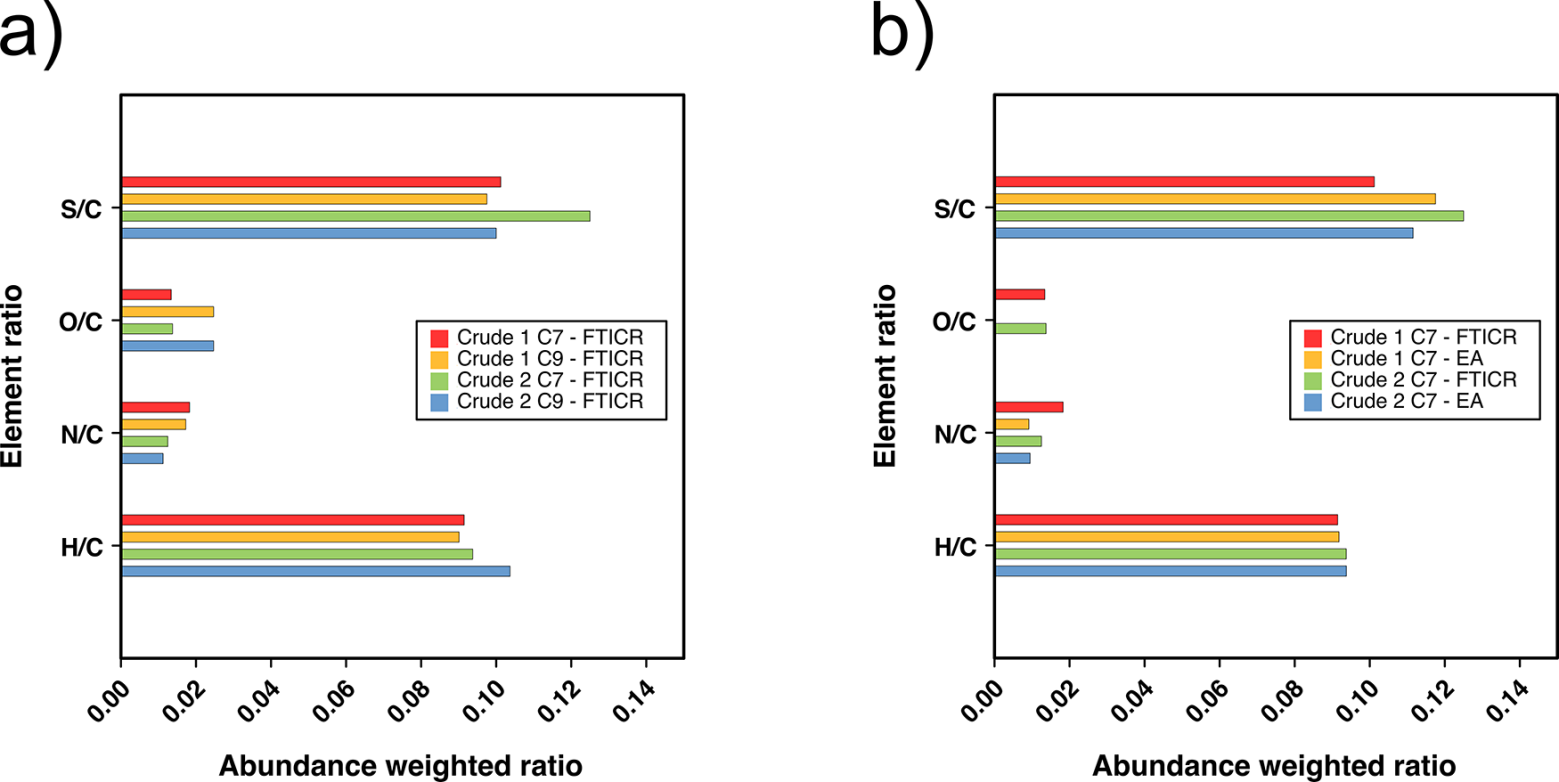
(a) Abundance-weighted elemental ratios as determined by FT-ICR MS for Crude 1 and Crude 2 asphaltenes, extracted using *n*-heptane and *n*-nonane. (b) Abundance-weighted elemental ratios for the *n*-heptane asphaltenes as calculated from FT-ICR MS data and determined by traditional elemental analysis.

To further understand the heteroatomic profiles of asphaltenes extracted from Crude 1 and Crude 2 using *n*-heptane and *n*-nonane, Figure 3 compares the abundance-weighted element ratios determined for the samples using different methods. Figure 3a shows the ratios for the four samples, as determined using the FT-ICR MS data (labeled as “FTICR”), and Figure 3b shows a comparison of the samples produced using *n*-heptane, which had been analyzed using both FT-ICR MS and elemental analysis (labeled “EA”). When comparing the results of the FT-ICR MS analyses and the elemental analysis, it is worth remembering that the FT-ICR MS data represent use of only one ionization method, operated only in positive-ion mode; despite this, the comparison shows similar values. According to the FT-ICR MS results, the asphaltenes extracted using *n*-heptane had slightly greater S/C and N/C ratios but significantly lower O/C ratios, when compared to asphaltenes extracted by *n*-nonane. This is consistent with the compound class distributions shown in Figure 1. Moreover, Crude 2 was determined to have higher S/C ratios compared to Crude 1, which is in agreement with the data shown in Table 1. The greater N/C contributions observed for Crude 1 are consistent with the more pronounced N_*n*_ and N_*n*_[H] classes observed in Figure 1, for example. Figure 3b shows a comparison of the FT-ICR MS data with the traditional elemental analyses of Crude 1 and Crude 2 asphaltenes obtained by *n*-heptane. It can be seen that Crude 2 has higher S/C ratios, in line with Figure 1. The elemental analysis provided data for C, H, N, and S contributions, and therefore, the O/C ratio was only provided by the FT-ICR data here. Finally, Crude 2 exhibits higher H/C ratios when compared to Crude 1, indicating a higher degree of aromaticity overall, as also shown in Table S1.

**Figure 4:**
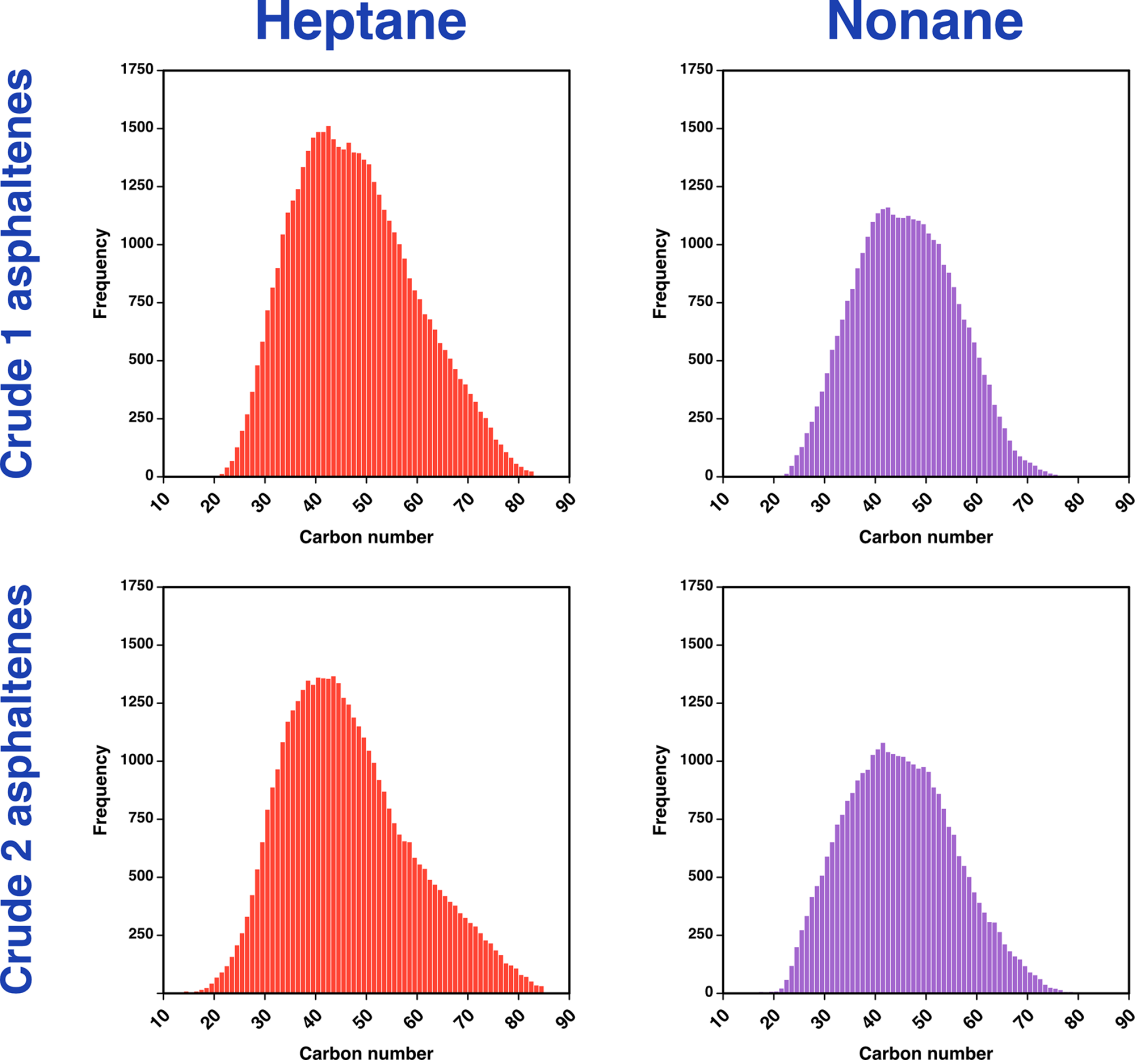
Plots of the count of number of components for each carbon number across all classes for the Crude 1 (top) and Crude 2 (bottom) asphaltenes, produced by extraction with *n*-heptane, in red, or *n*-nonane, in purple. The differences in the number of species observed for *n*-heptane vs *n*-nonane can be observed, as can the differences in the carbon number distribution.

To examine the effects upon mass range and number of components observed, the numbers of assigned molecular formulas across all heteroatom classes were categorized by carbon number and summed. Figure 4 shows carbon number distributions for all assignments for each of the four samples, based upon the FT-ICR MS data. Extraction using *n*-heptane resulted in a greater number of molecular formulas being assigned for each sample, following on from the summed signal intensities being higher on a compound class basis, as shown earlier. A wider carbon number range is also observed when using *n*-heptane; in addition to species of a lower carbon number being detected, Figure 4 highlights a less symmetrical distribution, with the more pronounced tail at the higher mass end and both samples extending to higher carbon number. Thus, the use of *n*-heptane is advantageous for the detection of the heavier components, compared to profiles measured following extraction using *n*-nonane.

**Figure 5:**
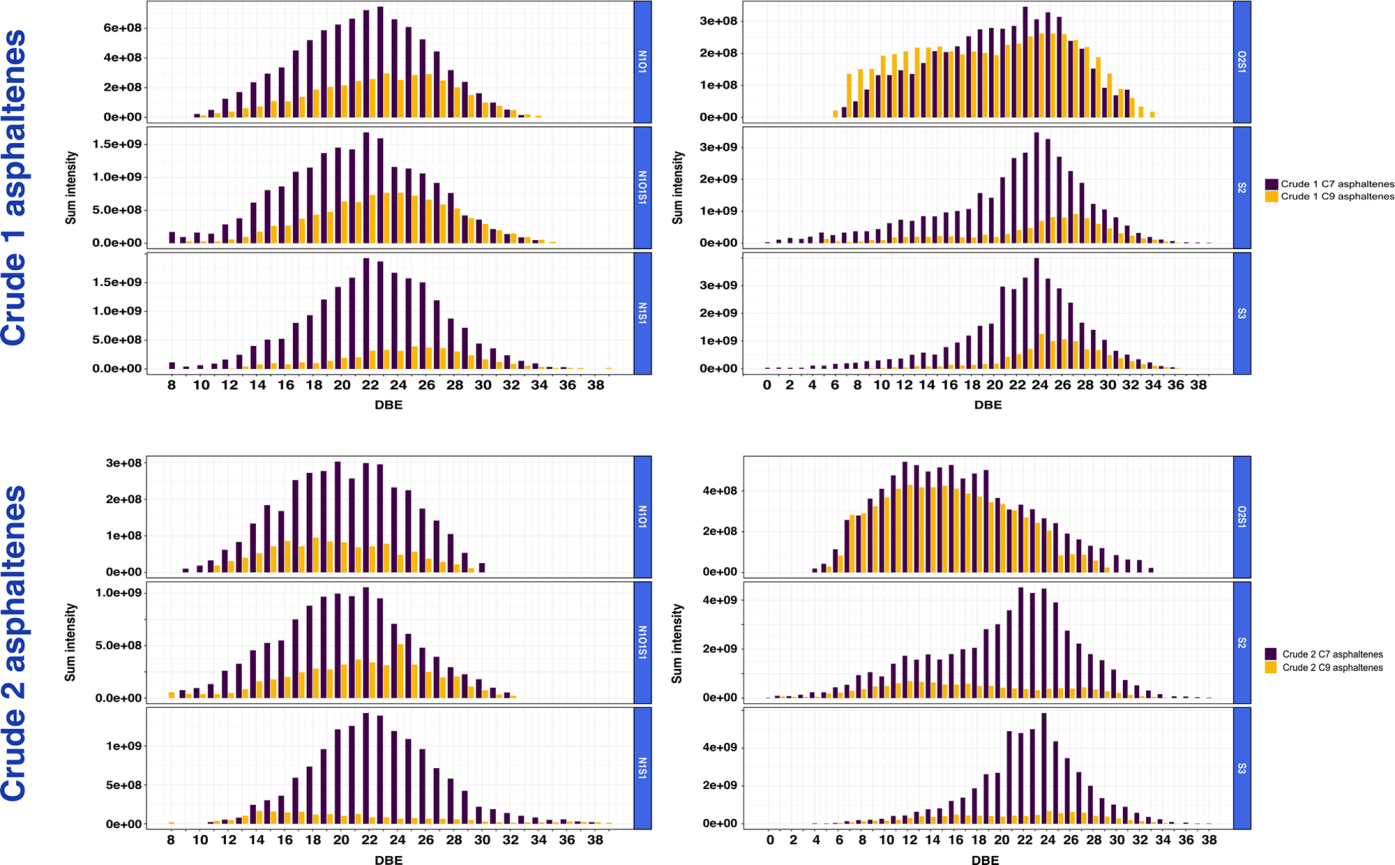
Plots of intensities for homologous series, summing intensities of the DBE values across all carbon numbers, for six selected classes (NO, NOS, NS, O_2_S, S_2_, and S_3_) of the asphaltenes separated from Crude 1 and Crude 2 investigated by positive-ion mode APPI.

The summed signal intensities of the DBE series for six selected compound classes present in asphaltenes acquired from Crude 1 and from Crude 2 are provided in Figure 5. Across all compound classes, the signals for the asphaltene components extracted using *n*-heptane are shown in Figure 5 to be more abundant than those of *n*-nonane. Moreover, the solvents access compounds of differing aromaticities, as the S_2_, S_3_, NOS, and NS components extend to lower DBE values when extracted using *n*-heptane, rather than *n*-nonane. Furthermore, the centers of the DBE distributions are offset to higher values when using *n*-nonane, primarily revealing the most unsaturated compositions in the NO, NOS, and NS classes. Uniquely, O_2_S shows different tendencies, where the extraction using *n*-nonane is targeting low- and high-DBE components with greater extent, offering more access to constituents with lower and higher DBE numbers, Furthermore, the extraction using *n*-heptane reveals a greater contribution to the middle range DBE species. The homologous series intensities for asphaltenes separated from Crude 2, as displayed in Figure 5, mostly show similar trends for both solvents. Showing a similar trend for Crude 2 as for Crude 1, the use of *n*-heptane resulted in the stronger signal for the NO, S_2_, S_3_, NOS, and NS classes. For the NOS class, the use of *n*-nonane primarily favors observation of species of higher DBE values, whereas the use of *n*-heptane also reveals the lower DBE constituents. Additionally, extraction using *n*-heptane enhances detection of higher DBE components in the S_2_ and S_3_ classes. The O_2_S class distinctively exhibits intense homologous series in different DBE ranges when comparing extracts produced using the two solvents. Generally, extracts produced using *n*-nonane span a narrower range of DBEs, and the mass spectra exhibit lower signal intensity, compared to extracts produced using *n*-heptane.

It can be deduced from Figure 5 that there are subtle compositional differences between asphaltenes generated from both Crude 1 and Crude 2, such as the higher prevalence of the lower DBE series for the sulfur-containing classes such as the S_2_, S_3_, NOS, and NS. Patterns within the DBE distribution for sulfur-containing classes provide potential structural insights. As an example, the S_2_ class exhibits homologous series that are more pronounced for DBEs 19, 21, and 24. For island-type structures with single, cata-condensed cores and incorporating two sulfur atoms, DBEs of 21 and 24 can result from a single thiophene group and additional aromatic rings or from two thiophene groups and at least one alicyclic ring (in addition to further aromatic rings); a DBE of 19 can result from one thiophene group and at least one alicyclic ring or two thiophene groups and at least two alicyclic rings. Examples of possible molecules with 19, 21, and 24 DBEs are shown in Figures S1–S3 in the Supporting Information. For Crude 1, the NS and NOS classes both begin at a DBE of 8. Pyrrolic and thiophenic compounds both prefer to form radical ions, rather than protonated species, when ionized by APPI; other structural motifs can result in different ionization behaviors. For example, nitrogen incorporated into a pyridinic group^47^ would lead to preferentially forming protonated species, by contrast. A cata-condensed island-type structure consisting of one pyrrolic group, one thiophenic group, and one six-membered aromatic ring would lead to a DBE of 8 and thus is a probable candidate. A DBE of 23 is also enhanced, which could represent the same base structure with the addition of further aromatic rings in a cata-condensed manner, while a DBE of 22 can result from a cata-condensed structure that includes at least two alicyclic rings or from a peri-condensed structure. For Crude 1, both the NS and NOS classes begin at the DBE of 8, and DBEs of 22 and 23 are prominent, potentially indicating that some of the species found within the two classes are related, such as through the oxidation of components of the NS class to produce the NOS class species.

DBE is used to address the degree of saturation of components within crude oil and other fractions. Shedding the light on the compositional profile of asphaltenes will greatly help in understanding of the structural motifs and aggregation behavior. Chacón-Patiño et al. utilized positive-ion APPI to effectively ionize a whole Petrophase 2017 asphaltene sample and reported that the DBE distribution was detected at the range of approximately 0–40. Moreover, they identified the distribution of South American Medium asphaltenes to be found within the DBE region of 15–35. In both samples, the most abundant species can be found in the DBE region of 20–25.^32^ Park and co-workers investigated a vacuum residue sample and were able to obtain asphaltenes using *n*-heptane and outlined the compositional space of asphaltenes observed in the DBE range of 0–55.^18^

**Figure 6:**
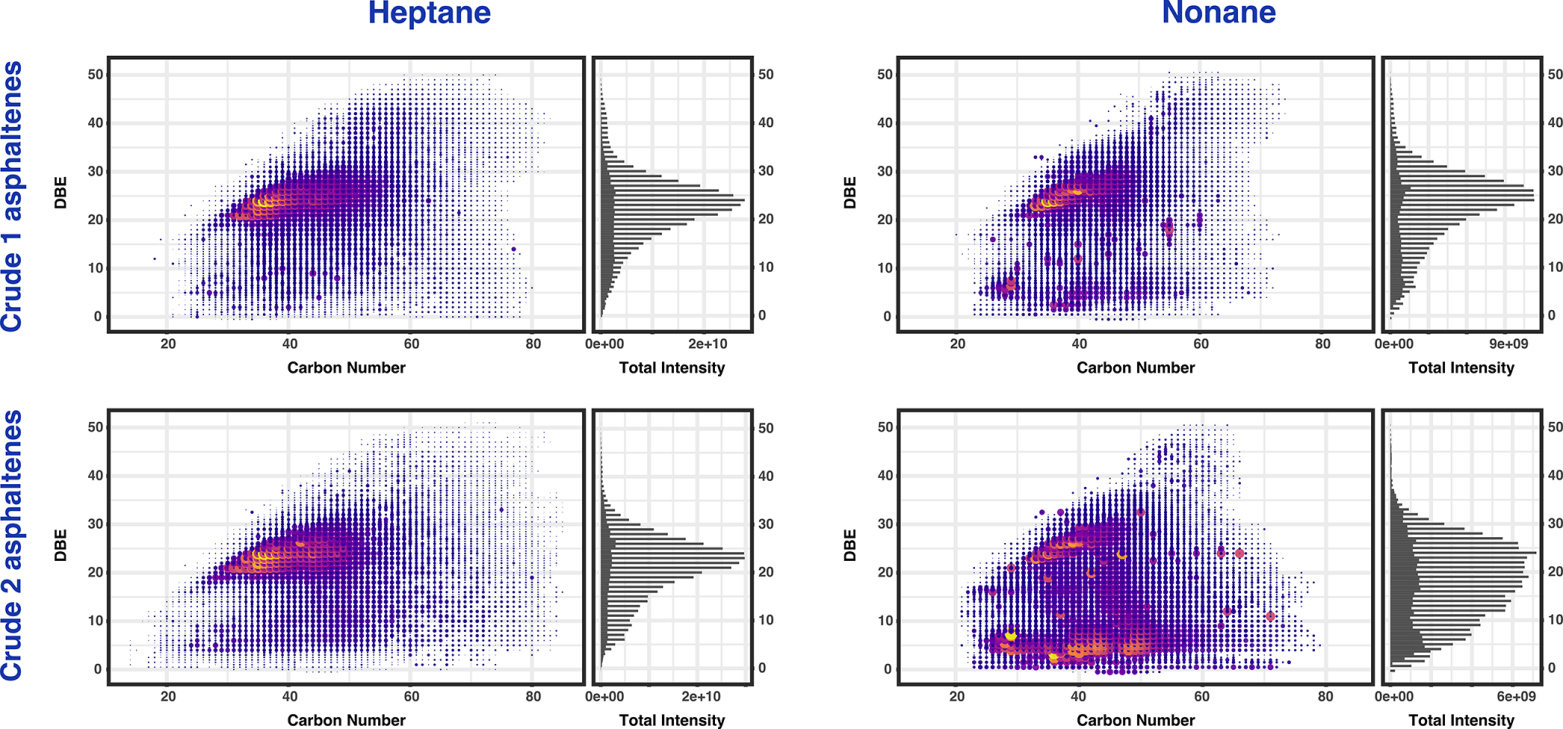
DBE vs carbon number profiles for all compound classes in the extracted asphaltenes of Crude 1 and Crude 2, with the sum of the DBE contributions from homologous series shown on the right-hand side for each plot.

Figure 6 illustrates the DBE distribution vs carbon number for classes contributing to the compositional profile of the extracted asphaltenes from Crude 1 and Crude 2 by Soxhlet extraction experiments along with total DBE intensities on the right. The DBE distribution of asphaltenes extracted by *n*-heptane spans a broad range of carbon numbers between 14 and 85 when compared to the *n*-nonane-extracted asphaltenes spanning the range of carbon numbers between 20 and 79. It is clear that *n*-heptane enhances the detection of species spanning a broader carbon number range and DBE range, in comparison to *n*-nonane-extracted asphaltenes, due to differences in solubility during extraction. Generally, the asphaltene samples exhibit similar scale of DBE intensities; however, asphaltenes extracted by *n*-heptane spanned over a greater range of carbon number ranges of approximately 30–56, while the asphaltenes extracted by *n*-nonane can be observed over the narrower carbon number range of 32–50. Moreover, *n*-heptane species exhibit one strongly abundant center of high DBE intensities, whereas the *n*-nonane components created several regions at lower DBE values with high DBE intensities targeting different DBE components, representing differences in selectivity when using the different solvents. While the extracts produced using *n*-heptane exhibited the typical profiles where there is a predominance of the high DBE and high carbon number species near the planar limit, typically ascribed to island-type asphaltenes, the asphaltenes extracted using *n*-nonane distinctively displayed a region of lower DBE species, particularly for the Crude 2 asphaltenes. This profile at lower DBE more closely resembles that expected for maltenes and may represent maltenes components that are less soluble in *n*-nonane, or it has also been stated that archipelago-type asphaltenes would be found in this region,^33^^51^. The differences in solubility in the alkanes of different lengths can be due not only to the polarity of the crude oil components but also the length and accessibility of the alkyl chains within the molecules (e.g., whether predominantly connecting cores of archipelago-type structures or on the outer edges of cores, particularly for island-type structures.

## Conclusions

Parallel separation of asphaltenes from two Middle Eastern petroleum samples was performed using two, different alkane-based solvents during Soxhlet extraction. This was followed by characterization using high-field FT-ICR mass spectrometry, which revealed differences during molecular characterization, and these should be considered when selecting a solvent during extraction.

The two asphaltene extracts, from crude oils of different origins from the same region, displayed similarities, but there were differences, such as the asphaltene extract of Crude 2 exhibiting a higher sulfur content than that of Crude 1. The choice of solvent influences the relative contributions of different heteroatom classes with *n*-heptane affording greater selectivity toward many nitrogen- and sulfur-containing classes.

When compiling the molecular formulas for all heteroatom classes according to carbon number, it was revealed that extraction using *n*-heptane led to observation of components across a broader mass range, and the distribution was less symmetrical, with a more pronounced tail at higher carbon number. Furthermore, extraction using *n*-heptane led to observation of the greatest number of components: more than 65 000 unique molecular formulas for Crude 1 asphaltenes and nearly 62 000 unique molecular formulas for the Crude 2 asphaltenes. When using *n*-nonane, these numbers were significantly lower at more than 48 000 and more than 43 000, respectively.

There were also differences in the DBE ranges observed, with *n*-heptane providing access to a broader DBE range for the two samples and also leading to greater contributions at the higher DBE (more aromatic) end. When examining plots of DBE versus carbon number, the diagonal band of data points at higher DBE for all extracts is where island-type asphaltenes would be expected, while the lower DBE region that is much more prevalent for the *n*-nonane extracts can represent maltenes that are less soluble in *n*-nonane, and it has also been stated that archipelago-type asphaltenes would be found in this region.

## Supporting Information Available

Averaged aromaticity index and double bond equivalents values for each sample (Table S1). Examples of structures to support discussion (Figures S1–S3) PDF)
