# Supplementary material for: Investigating the Influence of n-Heptane versus n-Nonane upon the Extraction of Asphaltenes
Source: Energy Fuels. 2022 Aug 1;36(16):8663–73. doi: 10.1021/acs.energyfuels.2c01168 (PMC9393859; doi:10.1021/acs.energyfuels.2c01168)
Supplement: Supplementary file 1 — ef2c01168_si_001.pdf [file ef2c01168_si_001.pdf]

Supporting Information

Investigating the Influence of n-Heptane versus n-Nonane upon the Extraction of Asphaltenes

Latifa K. Alostad [a], Diana Catalina Palacio Lozano [a], Benedict Gannon [a],

*Rory P. Downham [a], Hugh E. Jones [a, b], and Mark P. Barrow [*a]*

[a] Department of Chemistry, University of Warwick, Coventry, CV4 7AL, United Kingdom

[b] Molecular Analytical Sciences Centre for Doctoral Training, University of Warwick, Coventry, CV4 7AL, United Kingdom

[*]Corresponding author: M.P.Barrow@warwick.ac.uk

Special Issue dedicated to Oliver C. Mullins

Sample	AI_{AVG}	$AI_{MOD,AVG}$	DBE_{AVG}
**Crude 1 C7 asphaltenes**	0.41	0.41	20.8
**Crude 1 C9 asphaltenes**	0.44	0.44	21.3
**Crude 2 C7 asphaltenes**	0.40	0.40	19.8
**Crude 2 C9 asphaltenes**	0.38	0.39	18.2

Table S1: Averaged values for aromaticity index (AI), modified aromaticity index (AI_{mod}), and double bond equivalents (DBE) for Crude 1 n-heptane extracted asphaltenes, Crude 1 n-nonane extracted asphaltenes, Crude 2 n-heptane extracted asphaltenes, and Crude 2 n-nonane extracted asphaltenes

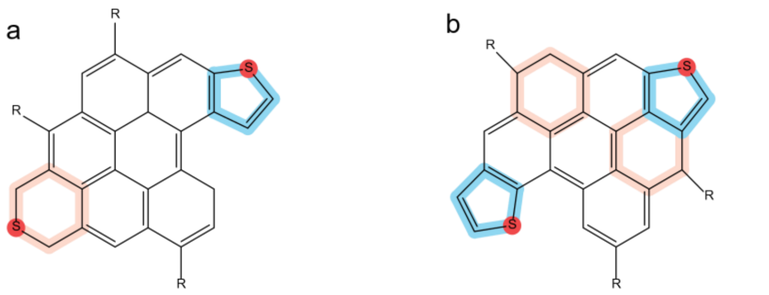

Figure S1: Examples of possible asphaltene structures with DBE values of 19. Left: asphaltene structure with a single thiophene group. Right: asphaltene structure with two thiophene groups.

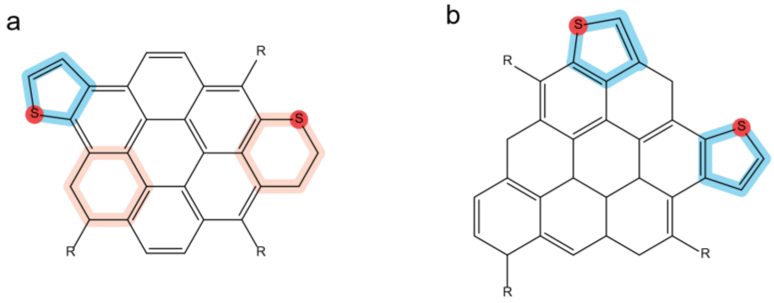

Figure S2: Examples of possible asphaltene structures with DBE values of 21. Left: asphaltene structure with a single thiophene group. Right: asphaltene structure with two thiophene groups.

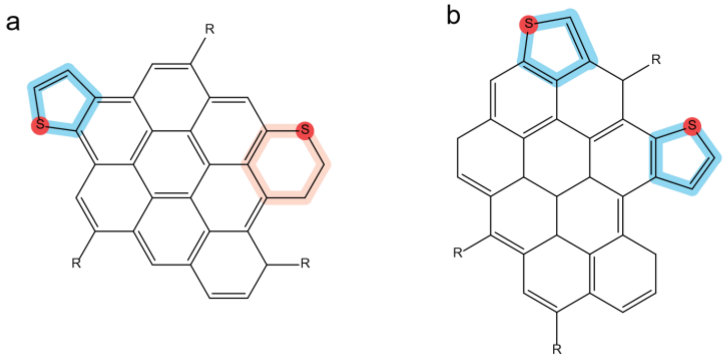

Figure S3: Examples of possible asphaltene structures with DBE values of 24. Left: asphaltene structure with a single thiophene group. Right: asphaltene structure with two thiophene groups.
